# Development of a Japanese Version of the Parenting Sense of Competence Scale, and Examining the Structure of Japanese Mothers’ Parenting Self-Efficacy

**DOI:** 10.3390/children11121460

**Published:** 2024-11-29

**Authors:** Tomomi Tanigo, Masayuki Endo, Kazutomo Ohashi

**Affiliations:** 1 Faculty of Nursing, Osaka Dental University, 11-8, Hanazono-machi, Kuzuha, Hirakata-shi 573-1121, Osaka, Japan; 2 Division of Health Science, Graduate School of Medicine, Osaka University, 1-7, Yamadaoka, Suita-shi 565-0871, Osaka, Japan; 3 Faculty of Global Nursing, Otemae University, 2-1-88, Otemae, Chuo-ku, Osaka-shi 540-0008, Osaka, Japan

**Keywords:** efficacy, mental competency, mother, parenting, personal satisfaction, postpartum period

## Abstract

Objective: Parenting self-efficacy (PSE) is an important factor in children’s development. Mothers’ PSE tends to be the lowest 1 month postpartum. A common measure of PSE is the Parenting Sense of Competence (PSOC) scale. However, no existing scale measures the PSE of Japanese mothers with newborns. Therefore, this study developed a Japanese version of the PSOC scale for mothers 1 month postpartum (Study 1) and investigated the structure of their PSE (Study 2). Methods: In Study 1, data were collected from mothers 1 month postpartum from April to October 2017, and an exploratory factor analysis was performed on their PSOC scores. In Study 2, data were collected from mothers 1 month postpartum from January to September 2022. A confirmatory factor analysis was conducted of the Japanese PSOC scale developed in Study 1 to investigate the structure of the participants’ PSE 1 month postpartum. Results: In Study 1, a 2-factor (Efficacy and Satisfaction) 12-item Japanese PSOC scale was obtained after deleting four items because of low factor loadings. In Study 2, the model showed an acceptable fit. The Japanese PSOC (12 items) had a moderate positive correlation with Rosenberg’s Self-Esteem Scale and the Maternal Attachment Inventory and a moderate negative correlation with the Edinburgh Postpartum Depression Scale. Furthermore, mothers whose children had siblings (versus no siblings) and mothers with three children (versus two children) had significantly higher PSOC scores. Conclusions: We developed a reliable and valid Japanese version of the PSOC for mothers 1 month postpartum and revealed the structure of their PSE.

## 1. Introduction

Parenting self-efficacy (PSE) refers to parents’ perceptions of their ability to perform tasks related to parenting [1]. As a dimension of Bandura’s broader self-efficacy theory [2] (pp. 190–196, 237–239), PSE reflects parents’ competence and confidence in raising children [3]. Many studies have explored the process through which a woman assumes the role of a mother [4,5] and [6] (pp. 38–51). In this process, a woman undergoes a transformation [7], in which she transitions from viewing herself primarily as a woman to identifying more with her role as a mother [8]. Women also acquire the ability to perform childcare tasks and increase their PSE [9].

There are also negative feelings associated with becoming a mother, such as anxiety about caring for children and difficulty raising them. In this study, we focused on mothers’ PSE, which has a positive, direct, and significant effect on their children’s health. Mothers with high PSE can manage their parenting responsibilities and feel a sense of accomplishment and satisfaction, even when faced with stressful situations [2] (pp. 237–239). Low PSE is associated with postpartum depression in parents, as well as adverse health outcomes and future problematic behavior in children [10]. Therefore, PSE is important for children’s health and development and for fostering positive parent-child relationships.

In Japan, nearly half of the population holds the traditional belief that men should be employed outside the home while women should manage household responsibilities [11]. Although the rate of men taking childcare leave is on the rise, husbands spend an average of 1 h and 23 min (with 49 min spent on childcare) on housework and childcare per day; these figures are low compared to international standards [12]. Research indicates that Japanese women experience “changing relationships with surrounding people” and “dissatisfaction with the husband’s involvement in childcare” as they acquire the role of a mother [13]. In many Asian countries, grandparents, fathers, and other relatives commonly work together to raise children. However, the situation in Japan is distinct; in a society with traditional values, Japanese mothers tend to raise their children alone. Given that the mother’s PSE directly affects her children’s health, it becomes imperative to examine the PSE of Japanese mothers.

The Parenting Sense of Competence (PSOC) scale is designed to measure PSE [14,15]. Initially developed by Gibaud-Wallston and Wandersman [16], it was adapted by Johnston and Mash [17]. The PSOC scale has English [9,17,18,19], Chinese [20], Thai [21], Portuguese [22], Polish [23], and Spanish versions [24], and is used worldwide. Abe and Kobayashi [25] developed a Japanese version of the PSOC scale for mothers with children up to 4 months of age. However, the factor structure of PSE among Japanese mothers has yet to be confirmed. Therefore, the PSE in Japanese mothers with newborns cannot be properly assessed.

Previous studies have demonstrated that educational interventions and parenting preparation programs increase PSE in primiparous women [26,27,28]. We aimed to accurately measure PSE in Japanese mothers using the PSOC scale and identify strategies to enhance their PSE. A previous study used the PSOC scale to longitudinally evaluate PSE in mothers from hospitalization to 8 months postpartum and found the lowest values occurred at 1 month postpartum [29]. Therefore, we developed a reliable and valid Japanese version of the PSOC scale for mothers 1 month postpartum (Study 1) and used the scale to examine the structure of their PSE (Study 2).

## 2. Materials and Methods

### 2.1. Study 1

#### 2.1.1. Procedure

We conducted an anonymous, self-administered questionnaire survey from April to October 2017 at an obstetric clinic in Osaka City, Japan, which has a population of over 2.69 million. At the 1-month postpartum checkup, the clinic staff explained the study to visiting mothers and provided them with a document that described its purpose. Consent was obtained if they completed the study questionnaire; therefore, submission of the survey questionnaire was confirmation of participants’ agreement to participate. The completed questionnaires were collected in a locked box. The sample size was determined as follows: number of items on the scale × 10 [30]. This study was approved by the Ethics Review Committee of Mukogawa Women’s University (approval number: 16–79; Approval date: 5 December 2016).

#### 2.1.2. Measures

The questionnaire comprised questions regarding the participants’ characteristics, the Japanese version of the PSOC scale (16 items), the Japanese version of Rosenberg’s Self-Esteem Scale (RSES), and the Japanese version of the Edinburgh Postpartum Depression Scale (EPDS). Participants’ characteristics included age, education level, employment status, household income, whether the woman was primiparous or multiparous, and the number of children the woman had.

The PSOC measures the degree of competence and confidence that parents feel when performing tasks related to childcare and development. Gibaud-Wallston and Wanderman [16] developed the original 17-item scale. However, Johnston and Mash [17] removed the item “Being a good mother/father is a reward in itself” because the factor loading was less than 0.4, resulting in a 16-item version of the scale. In the original study [16], the first factor explained 23.6% of the variance, the second factor explained 12.5%, and the two factors together explained 36.1% of the variance. In addition, internal consistency was used to assess reliability, and Cronbach’s alpha coefficients were reported. The alpha coefficient for the entire scale was 0.79, the Satisfaction factor was 0.75, and the Efficacy factor was 0.76. The overall score for the PSOC scale was negatively correlated with the Child Behavior Checklist (CBCL).

We obtained permission from Dr. Johnston, the original developer of the scale, to create the Japanese version. Researchers and English translators translated the scale into Japanese and backtranslated it, confirmed its consistency with the original text, and developed a Japanese version of the Parenting Sense of Competence Scale (Japanese PSOC scale, 16 items). The respondents rated all 16 items on a six-point Likert scale ranging from 1 (“strongly disagree”) to 6 (“strongly agree”). The total score ranged from 16 to 96 points, with higher scores indicating greater PES.

The Japanese version of the RSES and EPDS were used to assess the convergent validity of the Japanese PSOC scale. The Japanese version of the RSES is a 10-item scale that measures self-esteem [31]. The respondents rated the items on a four-point Likert scale ranging from 1 (“strongly disagree”) to 4 (“strongly agree”). The higher the score, the higher the self-esteem. The Japanese version of the RSES was developed by Yamamoto et al. [32] and has been used in many studies. Previous research has demonstrated that the higher the self-esteem, the higher the positive feelings regarding childcare [20].

The Japanese version of the EPDS is a 10-item scale that measures postpartum depression [33]. This scale was developed by Okano et al. [34] and has been widely used as a screening tool for postpartum depression in Japan. Previous studies have confirmed that maternal depressive symptoms and the PSOC are related [20,35].

#### 2.1.3. Data Analysis

In total, 172 responses were collected. We excluded the responses of seven mothers with missing data on the Japanese PSOC scale, the response of one mother whose children were junior high school students and older, and the responses of three mothers whose children’s ages were unknown. We excluded the response of the mother whose children were junior high school students or older because parents’ feelings toward child-rearing differ when their children are junior high school age or older vs. when they are elementary school age or younger. After exclusion, we analyzed the responses of 161 mothers.

An exploratory factor analysis was performed on responses to the Japanese PSOC scale (16 items). For sample validity verification, the Kaiser–Meyer–Olkin (KMO) value was calculated, and Bartlett’s test of sphericity was performed. Kaiser and Rice’s criteria [36] were adopted for the KMO measure. To determine the number of factors, an exploratory factor analysis was conducted using promax rotation with principal factor extraction. Cronbach’s α coefficient of internal consistency was calculated to assess reliability. An α coefficient of 0.7 or greater was considered acceptable [37]. To assess convergent validity, we calculated Pearson’s correlation coefficients between scores on the PSOC and RSES and between scores on the PSOC and the EPDS. SPSS for Windows, version 28.0, was used to analyze the data.

### 2.2. Study 2

#### 2.2.1. Procedure

We conducted an anonymous, self-administered questionnaire survey from January to September 2022 at an obstetric clinic in Nishinomiya City, Japan, a city with a population of over 480,000. Nishinomiya City was selected because it is an urban area that differs from the city for Study 1. As in Study 1, we approached mothers at their 1-month postpartum checkup and described the study to them. Completed questionnaires were collected from mothers who consented to participate. In Study 2, we used the Maternal Attachment Inventory (MAI), in addition to RSES and the EPDS. Bowlby [38] posited that when mothers establish secure attachments with their children, it helps them acquire a basic sense of trust that acts as the foundation for stable interpersonal relationships. Therefore, it is important to evaluate mothers’ attachment to their infants when evaluating child-rearing at 1 month postpartum. This study was approved by the Ethics Review Committee of Mukogawa Women’s University (approval number: 21–97; Approval date: 23 December 2021).

#### 2.2.2. Measures

The questionnaire comprised questions regarding participants’ characteristics as well as the Japanese versions of the PSOC developed in Study 1 (12 items), RSES, the EPDS, and the MAI [39]. The questions regarding participant characteristics were the same as those included in Study 1. Developed by Müller [40], the MAI is a measure of maternal attachment to infants. The Japanese version of the MAI was developed by Nakajima [39]. The respondents rated 26 items on a scale ranging from 1 to 4. The total score ranges from 26 to 104 points, with higher scores indicating a stronger attachment to the infant. The reliability and validity of the Japanese version of the MAI have been verified. Consent for use of the MAI in the questionnaire was obtained from the author.

#### 2.2.3. Data Analysis

In total, 149 responses were collected. We excluded the responses of 12 mothers with missing data for the Japanese PSOC scale. We also excluded the responses of two mothers whose children were junior high school students or older, and the responses of six mothers whose children’s ages were unknown. Consequently, we analyzed the responses of 129 mothers.

A confirmatory factor analysis of the Japanese PSOC scale (12 items) was performed. The KMO measure was calculated for sample validity verification, as described in Study 1. The two-factor structure was examined using the maximum likelihood method. The model fit was determined using the relative chi-square/degree-of-freedom ratio (χ^2^/df), goodness-of-fit index (GFI), adjusted goodness-of-fit index (AGFI), comparative fit index (CFI), and root mean square error of approximation (RMSEA). According to Schermelleh-Engel et al. [41], a χ^2^/df of 3 or less denotes an acceptable fit and 2 or less denotes a good fit. A GFI of 0.9 or higher, an AGFI of 0.85 or higher, a CFI of 0.95 or higher, and an RMSEA between 0.05 and 0.08 denoted acceptable fit. Next, we calculated Pearson’s correlation coefficients between scores on the PSOC and scores on RSES, the EPDS, and the MAI to determine convergent validity. In addition, we examined whether the PSOC score differed significantly based on the presence or absence of siblings and the number of children, using unpaired *t*-tests. IBM SPSS Statistics for Windows, Version 28.0. and SPSS AMOS for Windows, Version 26.0, were used to analyze the data.

## 3. Results

### 3.1. Study 1

#### 3.1.1. Exploratory Factor Analysis of the Japanese PSOC Scale (16 Items)

Table 1 presents the characteristics of the participants. We compared these characteristics with the national average and found that the participants in this study were highly educated. However, their employment rate and household income were low. We confirmed that there were no outliers, as no values were more than three standard deviations away from the mean.

The KMO measure was 0.73, and Bartlett’s test of sphericity was significant, χ^2^ = 555.3, df = 120, and *p* < 0.001. Scores on the Japanese PSOC scale (16 items) averaged 55.6 (range: 30–77 points). Among the 16 items, no items had an average score of 6 or more (+1SD) or less than 1 (−1SD), indicating there were no ceiling or floor effects.

Exploratory factor analyses were performed of the Japanese PSOC scale (16 items) with factor extraction as the principal factor method and promax rotation. The initial solution contained five factors. However, only two factors were retained, which were the only factors that accounted for over 10% of the variance and were interpretable. A second analysis was conducted with the number of factors to extract set to two (Table 2). Four items (Items 5, 7, 8, and 12) were deleted due to their factor loadings being below 0.35. The third and final analysis was conducted with the remaining 12 items (Table 3), which comprise the Japanese PSOC scale. The cumulative variance explained was 34.1%.

Factor 1 of the Japanese PSOC scale (12 items) was named “Efficacy”, as it was related to the mother’s role and the effectiveness of childcare techniques. Factor 2 was named “Satisfaction”, as it was related to the sense of accomplishment from fulfilling the mother’s role and the fulfillment derived from childcare. The average score on the Japanese PSOC scale (12 items) was 44.0 (range: 25–59 points).

#### 3.1.2. Reliability

Cronbach’s α for the entire scale and for each factor exceeded 0.7 (Table 3).

#### 3.1.3. Convergent Validity

The Japanese PSOC scale (12 items) demonstrated convergent validity with RES and the EPDS. A moderate positive correlation was observed between Japanese PSOC scale scores and RSES scores (Table 4). Conversely, a moderate negative correlation was observed between Japanese PSOC scale scores (12 items) and EPDS scores.

### 3.2. Study 2

#### 3.2.1. Structural Analysis of the Japanese Version of the PSOC Scale (12 Items)

Table 1 presents the characteristics of the participants in Study 2. These participants were highly educated, had a low employment rate, and had a higher household income than the national average.

**Figure 1:**
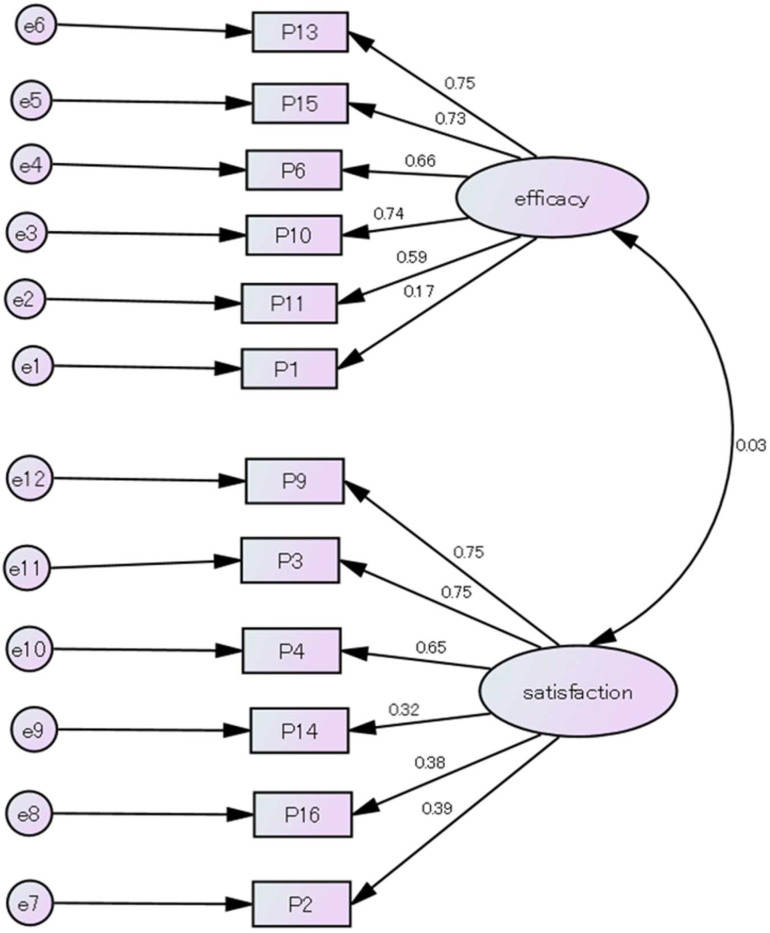
Confirmatory factor analysis of the Japanese PSOC scale (12 items). *Note.* CMIN = 87.99, df = 53, CMIN/df = 1.66, GFI = 0.907, AGFI = 0.864, CFI = 0.904, RMSEA = 0.072.

We confirmed that there were no outliers, as no values were more than three standard deviations away from the mean. The mean score on the Japanese PSOC scale (12 items) was 44.6 (range, 24–60). The KMO value was 0.77, and Bartlett’s test of sphericity was significant, χ^2^ = 415.3, df = 66, and *p* < 0.001. We conducted a confirmatory factor analysis of the Japanese PSOC scale (12 items) developed in Study 1 using the maximum likelihood estimation method. The path diagrams and model fit are shown in Figure 1.

#### 3.2.2. Relationships Between the Japanese PSOC Scale Scores and Scores on RSES, the EPDS, and the MAI

There was a moderate positive correlation between Japanese PSOC scale scores and RSES scores (r = 0.464, *p* < 0.01, *n* = 129). Japanese PSOC scale scores had a moderate negative correlation with EPDS scores (r = −0.393, *p* < 0.01, *n* = 129). Furthermore, there was a moderate positive correlation between Japanese PSOC scale scores and MAI scores (r = 0.416, *p* < 0.01, *n* = 128).

#### 3.2.3. Differences in the Scores on the Japanese PSOC Scale (12 Items) by the Presence or Absence of Siblings and the Number of Children

Japanese PSOC scale scores differed significantly between the sibling (*n* = 83) and non-sibling groups (*n* = 46), with the scores being significantly higher in the sibling group (45.8 vs. 42.6, *p* < 0.01). Regarding the number of children, there was no difference in the PSOC scale scores between mothers with one child (*n* = 46) and mothers with two children (*n* = 60) (48.5 vs. 50.5, *p* = 0.118). However, the PSOC scores differed significantly between mothers with two children (*n* = 60) and those with three children (*n* = 20). Mothers with three children had significantly higher PSOC scale scores (50.5 vs. 54.2, *p* < 0.01).

## 4. Discussion

### 4.1. Development of the Japanese PSOC Scale (12 Items) (In Study 1)

The Japanese PSOC scale comprises 12 items and was constructed with a bifactorial structure consisting of the dimensions of Satisfaction and Efficacy. The original version of the scale also had a two-factor structure consisting of the dimensions of Satisfaction and Efficacy [17]. A study involving Canadian parents showed a similar two-factor structure [19], as did a study involving Chinese mothers hospitalized postpartum [20]. Furthermore, a systematic review [10] reported the frequent use of the PSOC total score and the scores from the Satisfaction and Efficacy subscales. Therefore, this study confirmed the factor structure of the original scale and the results of previous studies.

In an exploratory factor analysis of the Japanese PSOC scale (12 items), the cumulative variance explained was 34.0%, which is similar to that reported in the original version (36.1%) [17]. Additionally, the Cronbach’s α coefficient for the entire scale and the two subscales indicated acceptable internal consistency [37]. Furthermore, Japanese PSOC scale scores showed a moderate positive correlation and moderate negative correlation with RSES and EPDS scores, respectively, indicating the reliability and convergent validity of the Japanese PSOC scale (12 items).

From the original version of the Japanese PSOC scale (12 items), we deleted Items 5, 7, 8, and 12 due to low factor loadings. Previous studies targeting mothers with newborns [20,25] also showed low factor loadings for Items 8 and 12. Although parents of children aged 4–9 years were recruited during the development of the original PSOC scale, it was difficult for mothers with newborns to rate Item 8 (“A difficult problem in being a parent is not knowing whether you are doing a good job or a bad one”) and Item 12 (“My talents and interests are in other areas, not in being a parent”). We believe the factor loadings were low because mothers with newborns have a life primarily centered on childcare, with little time or energy for other activities. Interestingly, Item 5 (“My mother was better prepared to be a good mother than I am”) and Item 7 (“Being a parent is manageable, and any problems are easily solved”) showed large factor loadings in the original PSOC scale [17]. The Chinese version [20], which targeted mothers with newborns, also showed large factor loadings. Hence, the low factor loadings for Items 5 and 7 were specific to the Japanese version and attributable to the characteristics of Japanese mothers. These results suggest that Japanese mothers tend not to compare their own parenting to their mothers’ parenting. They also tend not to think that it is easy to manage childcare or solve child-related problems.

### 4.2. Structural Analysis of PSE in Japanese Mothers 1 Month Postpartum (In Study 2)

For our confirmatory factor analysis of the Japanese PSOC scale (12 items), the CFI was 0.904 and not 0.95 or higher. However, χ^2^/df was ≤2, demonstrating a good fit. The GFI was 0.9 or higher, AGFI was 0.85 or higher, and RMSEA was between 0.05 and 0.08, suggesting an acceptable fit. Consequently, the Japanese version of the PSOC scale (12 items) had a two-factor structure of “Efficacy” and “Satisfaction”, similar to the original PSOC scale. In addition, the scores on the Japanese PSOC scale were moderately positively and moderately negatively correlated with RSES and EPDS scores, respectively, suggesting convergent validity for the scale.

### 4.3. Relationship Between the PSE and MAI Scores of Japanese Mothers 1 Month Postpartum and Relationship with the Number of Children (In Study 2)

There was a moderate correlation between PSE (assessed by the Japanese PSOC scale) and attachment to the infant (assessed by the MAI), suggesting that increased attachment increases mothers’ confidence in child-rearing. Systematic reviews of the factors associated with PSE have found relationships between parenting stress, maternal depression, and social support. However, the relationship between PSE and attachment has yet to be examined [45]. One study clarified that parental attachment to children and PSE are the basis for healthy child development [46]. A second study found that PSE and MAI increase when home visits are provided to parents raising children with cleft lip and palate [47]. A third study reported that stable attachment is the basis for a child to build good relationships with others [38]. These findings suggest that childcare support should be provided that considers the mother’s attachment to the infant at 1 month postpartum, as it will lead to improved PSE.

Japanese PSOC scale scores were significantly higher among mothers whose children had siblings than among mothers whose children had no siblings. This result is consistent with that of Ngai et al. [20], who conducted a study of Chinese mothers hospitalized after childbirth. From postpartum hospitalization to 8 months postpartum, a longitudinal study conducted at four time points (during postpartum hospitalization, 1 month postpartum, 4 months postpartum, and 8 months postpartum) revealed that PSOC scores remained consistently stable for mothers whose children had siblings. In contrast, mothers whose children did not have siblings exhibited lower scores during postpartum hospitalization compared to mothers whose children had siblings. At 1 month postpartum, the scores were lower. However, at 4 and 8 months postpartum, scores increased for mothers whose children had no siblings and surpassed those of mothers whose children had siblings [48]. In the current study, mothers with three children scored significantly higher on the PSOC scale compared to mothers with two children. We attribute this difference to the increased experience that mothers of three children gained in managing the demands of caring for multiple children simultaneously.

Overall, the findings suggest that child-rearing experiences improve mothers’ PSE. Interventions exist for improving the PSE of primiparous women, and their effectiveness has been verified [26,27,28]. However, interventions for multiparous women have not been sufficiently developed. Providing support that considers the mother’s child-rearing experience may be effective in improving mothers’ PSE.

### 4.4. Implications for Children’s Health

This study’s findings indicated that mothers’ PSE at 1 month postpartum was associated with postpartum depression, self-esteem, and attachment to their child. By fostering a healthy attachment relationship between the mother and child during infancy, particularly 1 month after birth, the child can develop a fundamental sense of security that serves as the foundation for future interpersonal connections. Therefore, PSE has a significant impact on the healthy development of children.

With the development of the Japanese version of the PSOC scale (12 items), it is possible to not only measure the PSE of Japanese mothers 1 month postpartum but also to make comparisons across countries. Additionally, our scale can be used to evaluate care tailored to the mother’s level of parenting experience and attachment to the child and can be used internationally.

Future research is needed to clarify the relationships between mothers’ PSE and feelings of isolation (UCLA Loneliness Scale) and husbands’ participation in childcare, by considering the lonely childcare environment unique to Japanese people.

Moreover, future research should guide the development and evaluation of support methods that are tailored to mothers’ child-rearing experiences and foster healthy attachments between mothers and their infants.

### 4.5. Limitations

Our study had two limitations. First, an adequate sample of average Japanese mothers could not be obtained, even though Studies 1 and 2 were conducted in different regions of Japan. Our results were obtained from a group with a higher educational background than the national average, which limits the generalizability of the findings. Future studies should conduct a large-scale survey using the PSOC scale. Second, the methods used to verify the validity and reliability of the PSOC scale were limited. In addition to the methods used here, composite reliability or McDonald’s Omega is used to verify reliability, and mean variance is used to verify validity. Such methods must be used to investigate validity and reliability for higher-quality scale development.

## 5. Conclusions

We developed a reliable and valid Japanese version of the PSOC scale for mothers 1 month postpartum with 12 items and two factors (efficacy and satisfaction). We performed confirmatory factor analysis with different subjects, and the model fit was acceptable. Japanese mothers’ PSE at 1 month postpartum was found to be correlated with their attachment to their infants. Additionally, mothers whose children had siblings (versus mothers whose children had no siblings) and mothers with three children (versus those with two children) reported higher PSOC scores. These results suggest that care that promotes a healthy attachment to the infant and considers the mother’s childcare experience may improve mothers’ PSE, which will positively affect children’s health.

## Figures and Tables

**Table 1:** Participants’ characteristics, RSES scores, and EPDS scores.

	Study 1 (*n* = 161)	Study 2 (*n* = 129)	National Data
Mean (SD) age (in years)	31.8 (4.7)	32.8 (4.3)	
Primiparous/Multiparous *n* (%)			
Primiparous	76 (47.2)	46 (35.7)	45.9% ^1^
Multiparous	85 (52.8)	83 (64.3)	
Education level *n* (%)			
Until high school	42 (26.3) ^2^	8 (6.2)	32.0% ^3^
Junior college/university or above	118 (73.8) ^2^	121 (93.8)	
Employment status *n* (%)			
Unemployed	86 (39.1)	48 (37.2)	24.1% ^4^
Employed	75 (60.9)	81 (62.8)	75.9%
Household income *n* (%)			
Less than JPY 5 million	63 (41.4) ^5^	21 (17.4) ^6^	22.2% ^4^
JPY 5 million or more	89 (58.6) ^5^	100 (82.6) ^6^	77.8%
Mean (SD) score on RSES	34.3 (6.7)	36.3 (6.1) ^7^	
Mean (SD) score on the EPDS	6.6 (4.6)	4.6 (4.0)	

^1^ Ministry of Health, Labour and Welfare of Japan [42], ^2^ *n* = 160, ^3^ Calculated from national census results [43] (30–34 year old women 2022), ^4^ Comprehensive Survey of Living Conditions [44] (Families of Children 2021), ^5^ *n* = 152, ^6^ *n* = 121, ^7^ *n* = 128, RSES: Rosenberg’s Self-Esteem Scale, EPDS: Edinburgh Postpartum Depression Scale, SD: Standard deviation.

**Table 2:** Factor loadings of the exploratory factor analysis of the Japanese PSOC scale (16 items).

Item	Factor 1	Factor 2
1. The problems of taking care of a child are easy to solve once you know how your actions affect your child, an understanding I have acquired.	**0.468**	−0.085
2. Even though being a parent could be rewarding, I am frustrated now while my child is at his/her present age.	0.121	**0.386**
3. I go to bed the same way I wake up in the morning—feeling like I have not accomplished a whole lot.	−0.011	**0.648**
4. I do not know what it is, but sometimes, when I am supposed to be in control, I feel more like the one being manipulated.	−0.048	**0.536**
5. My mother was better prepared to be a good mother than I am.	−0.063	−0.067
6. I would make a fine model for a new mother to follow to learn what she would need to know to be a good parent.	**0.686**	0.04
7. Being a parent is manageable, and any problems are easily solved.	0.306	0.099
8. A difficult problem in being a parent is not knowing whether you are doing a good job or a bad one.	0.263	0.276
9. Sometimes, I feel like I am not getting anything done.	0.094	**0.722**
10. I meet my own personal expectations for expertise in caring for my child.	**0.584**	0.049
11. If anyone can find the answer to what is troubling my child, I am the one.	**0.474**	−0.084
12. My talents and interests are in other areas, not in being a parent.	−0.061	0.222
13. Considering how long I have been a mother, I feel thoroughly familiar with this role.	**0.699**	−0.018
14. If being a mother of a child was more interesting, I would be motivated to do a better job as a parent.	−0.178	**0.473**
15. I honestly believe that I have all the skills necessary to be a good mother to my child.	**0.707**	−0.23
16. Being a parent makes me tense and anxious.	0.117	**0.426**

*Note*. The bold text indicates items with factor loadings greater than 0.35. PSOC: Parenting Sense of Competence.

**Table 3:** Factor loadings of the exploratory factor analysis of the Japanese PSOC scale (12 items).

Item	Factor	
	Efficacy	Satisfaction
15	**0.703**	−0.178
13	**0.697**	0.011
6	**0.672**	0.08
10	**0.562**	0.074
11	**0.473**	−0.087
1	**0.394**	0.146
9	0.067	**0.748**
3	−0.009	**0.651**
4	−0.054	**0.556**
14	−0.18	**0.472**
16	0.069	**0.411**
2	0.104	**0.396**
Contribution rate (%)	22.353	11.728
Cumulative contribution rate (%)	22.353	34.08
Cronbach’s α coefficient	0.739	
	0.752	0.703

*Note*. The bold text indicates items with factor loadings greater than 0.35.

**Table 4:** Correlation between scores on the Japanese PSOC scale (12 items) and scores on the RSES and EPDS.

	RSES	EPDS
PSOC total scale	0.454 **	−0.515 **
PSOC efficacy subscale	0.349 **	−0.234 **
PSOC satisfaction subscale	0.360 **	−0.556 **

** *p* < 0.001.

## Data Availability

The data presented in this study are available on request from the corresponding author due to privacy and ethical restrictions.
